# Expression and functional characterization of three peptidoglycan recognition proteins, PGRP-L1, PGRP-L2 and PGRP-S in snakehead (*Channa argus*)

**DOI:** 10.1016/j.cirep.2025.200248

**Published:** 2025-09-04

**Authors:** Zhen Yu Zhou, Wei Hao, Pin Nie, Shan Nan Chen, Jing Hou

**Affiliations:** a School of Marine Science and Engineering, Qingdao Agricultural University, Qingdao, Shandong Province 266109, China; b Laboratory for Marine Biology and Biotechnology, Qingdao National Laboratory for Marine Science and Technology, Qingdao, Shandong Province 266237, China; c State Key Laboratory of Breeding Biotechnology and Sustainable Aquaculture, Institute of Hydrobiology, Chinese Academy of Sciences, Wuhan 430072, China

**Keywords:** *Channa argus*, PGRP, PGN, Amidase activity, Antibacterial activity, NF-κB

## Abstract

Peptidoglycan recognition proteins (PGRPs) are evolutionarily conserved pattern recognition receptors critical for antibacterial immunity across vertebrates and invertebrates. In this study, two long-type (CaPGRP-L1, -L2) and one short-type (CaPGRP-S) PGRP genes were identified in snakehead (*Channa argus*), an economically important aquaculture species. Sequence analysis revealed that all three PGRPs contain a conserved Zn²⁺-binding site (composed of two His, one Tyr, and one Cys), essential for amidase activity. Tissue distribution profiling showed distinct expression patterns, with high transcript levels detected in liver, intestine or gill. Functional assays demonstrated that recombinant CaPGRP-L1, -L2, and -S bound peptidoglycan (PGN), displayed Zn²⁺-dependent amidase activity, and inhibited *Aeromonas hydrophila* infection. Furthermore, three PGRPs activated NF-κB promoter reporter plasmid in a dose-dependent manner, while NF-κB subunits c-Rel and p65 upregulated the transcriptional activity of CaPGRP-L1, -L2, and -S luciferase promoters, suggesting that snakehead PGRPs are involved in NF-κB signaling. Collectively, this study has explored the function of snakehead PGRPs, and deepens our understanding on innate immune mechanisms in fish, which should provide a theoretical foundation for the application of snakehead PGRPs in preventing and controlling diseases in aquaculture.

## Highlights

• Two long-type PGRP and one short PGRP, named as CaPGRP-L1, -L2 and -S were identified from snakehead.
• CaPGRP-L1, -L2 showed high expression in the intestine and liver, while CaPGRP-S was abundant in the gill.
• Three snakehead PGRPs demonstrated PGN-binding activity, amidase activity and antibacterial activity.
• Three snakehead PGRPs are involved in NF-κB signaling.

## Introduction

Peptidoglycan recognition proteins (PGRPs) are a class of innate immune molecules that play a pivotal role in responding to bacterial infection by binding to peptidoglycan (PGN), a key component of bacterial cell walls [1]. These proteins are evolutionarily conserved across vertebrates and invertebrates, including insects, mammals, amphibians, and fish [2,3]. Since their initial discovery in the hemolymph of silkworms in 1996, PGRPs have been extensively studied in various organisms [4].

Over the past decades, significant progress has been made in elucidating the expression patterns, structural characteristics, antimicrobial functions, and immunoregulatory roles of PGRPs across diverse species. In fruit fly (*Drosophila melanogaster*), PGRPs are classified into two main categories based on the length of their transcripts [5,6]. The short transcripts include PGRP-SA, -SB1, -SB2, -SC1A, -SC1B, -SC2, and -SD, while long transcripts include PGRP-LA, -LB, -LC, -LD, and -LE [7,8]. Notably, PGRP-LA, -LC and -LE activate the immune deficiency (IMD) pathway upon Gram-negative bacterial detection, while PGRP-SA and -SD trigger the Toll pathway in response to Gram-positive bacterial and fungal infections [5,8,9]. Furthermore, both the IMD and Toll pathways ultimately activate NF-κB transcription factors, thereby leading to the production of antimicrobial peptides (AMPs) [2,6,9]. Intriguingly, NF-κB can also activate PGRP-S transcription in mice by binding to κB motifs in its promoter region [10]. Mammals possess four PGRPs, designated as PGLYRP-1, PGLYRP-2, PGLYRP-3, and PGLYRP-4 [11]. Among these, PGLYRP-2 exhibits amidase activity capable of hydrolyzing bacterial peptidoglycan through cleavage of the lactyl bond between N-acetylmuramic acid (NAM) and the stem peptide [12]. Functionally, PGLYRP-2 can not only neutralize bacterial threats but also modulate immune responses by attenuating pro-inflammatory signaling [12,13].

To date, PGRPs have been identified in several species of fish, including zebrafish (*Danio rerio*) [14], rockfish (*Sebastes schlegelii*) [15], large yellow croaker (*Larimichthys crocea*) [16], rainbow trout (*Oncorhynchus mykiss*) [17], common carp (*Cyprinus carpio*) [18,19], grass carp (*Ctenopharyngodon idella*) [20], Nile tilapia (*Oreochromis niloticus*) [21], orange-spotted grouper (*Epinephelus coioides*) [22,23], sea bass (*Lateolabrax maculatus*) [24], largemouth bass (*Micropterus salmoides*) [25], and great blue-spotted mudskipper (*Boleophthalmus pectinirostris*) [26]. Fish PGRP-2, belonging to the long-type PGRP (PGRP-L), is homologous to mammalian PGLYRP-2 and has been demonstrated to exhibit both antibacterial and amidase activities [27]. In contrast, PGRP-5 (also termed PGRP-S) and PGRP6 (also termed PGRP-L), which are unique to fish and absent in mammals, are considered to have both peptidoglycan-lytic amidase activity and antibacterial activity [27]. In addition, PGRPs function as multifunctional regulators, influencing critical pathways like TLR signaling, developmental control, and apoptosis in zebrafish [28,29].

In general, PGRPs are essential components of the innate immune system, with diverse roles in pathogen recognition, immune regulation, and host defense. Given their importance, targeted research in farmed fish species is critical for developing effective disease prevention strategies. For instance, common carp PGRP2 demonstrates multifaceted protective functions including antibacterial activity, bacterial agglutination, intestinal mucosal protection, and enhanced resistance to *Aeromonas hydrophila* infection [19]. Similarly, large yellow croaker PGRP5 enhances innate immunity by promoting macrophage phagocytosis against *Staphylococcus aureus* [30]. However, PGRPs have not yet been studied in snakehead (*Channa argus*), a commercially important freshwater aquaculture species in Asia that suffers significant economic losses due to bacterial and viral pathogens [[31], [32], [33], [34]]. Therefore, in current study, three PGRP genes, designed as CaPGRP-L1, CaPGRP-L2 and CaPGRP-S, were cloned and identified in snakehead. Their roles in PGN recognition, amidase activity, NF-κB signaling activation and antibacterial activity were systematically investigated. We further demonstrated that the proximal promoters of these genes are activated by NF-κB transcription factors, including RelB, c-Rel, and p56. Phylogenetic and functional analyses revealed that caPGRP-L1, -L2 and -S are evolutionarily conserved in teleost fish. The present results advance our understanding on PGRP functions in fish immunity and provide theoretical basis for their potential application in disease management for snakehead aquaculture.

## Materials and methods

### Fish, cell line and bacterial strain

Healthy snakehead (*Channa argus*), averaging 200 ± 20 g in weight, were obtained from a fish farm in Jining, Shandong Province, China. Human Embryonic Kidney 293T (HEK293T) cells were maintained in Dulbecco’s modified Eagle’s medium (DMEM, Gibco) supplemented with 10 % fetal bovine serum (FBS) and incubated at 37 °C with 5 % CO₂ (Thermo Fisher). Epithelioma papulosum cyprini (EPC) cells were cultured in Medium 199 (M199, Gibco) containing 10 % FBS at 28 °C [35]. *Aeromonas hydrophila* was grown in tryptic soy broth (TSB) medium at 30 °C, and bacterial concentrations were determined by spectrophotometry at OD_540_. All animal experiments were conducted in compliance with the national general guidelines in terms of laboratory animal care and use, and approved by the school and university.

### Bioinformatics analysis

The complete open reading frame (ORF) of snakehead PGRPs cDNA was determined using the NCBI ORF Finder tool (https://www.ncbi.nlm.nih.gov/orffinder/). For sequence homology analysis, we performed BLAST searches against the NCBI database (https://blast.ncbi.nlm.nih.gov/Blast.cgi). The deduced amino acid sequence was carried out using the ExPASy proteomics tools (http://expasy.org/tools/), while protein domain prediction was conducted with the NCBI Conserved Domain Search service (https://www.ncbi.nlm.nih.gov/Structure/cdd/wrpsb.cgi) and Simple Modular Architecture Research Tool (SMART) (https://smart.embl-heidelberg.de/). To establish evolutionary relationships, a phylogenetic tree was constructed using the Neighbor-Joining (NJ) algorithm based on the deduced amino acid sequences in MEGA X software, with branch support assessed by 2000 bootstrap replicates. The accession numbers of all snakehead PGRP genes included in this study were provided in Supplementary Table 1. Potential NF-κB binding sites in the promoter regions of snakehead PGRPs were predicted using the JASPAR database (https://jaspar.elixir.no/) and subsequently validated through manual analysis.

### Cloning of CaPGRP-L1, CaPGRP-L2 and CaPGRP-S

Total RNA was isolated from the gill, intestine, and liver of snakehead using TRIzol reagent (Invitrogen), following the manufacturer’s protocol. First-strand cDNA was synthesized from the extracted RNA using the RevertAid™ First Strand cDNA Synthesis Kit (Thermo Scientific). The full-length open reading frames (ORFs) of CaPGRP-L1, -L2 and -S were amplified by PCR with gene-specific primers designed according to their reference sequences (EXN66_Car003170, EXN66_Car016280 and EXN66_Car008655) from snakehead genome (GCA_004786185.1). The PCR conditions were as follows: initial denaturation at 95 °C for 3 min, 30 cycles of 95 °C for 35 s, 55 °C for 35 s, and 72 °C for 60 s, followed by a final extension at 72 °C for 10 min. The resulting PCR products were cloned into the pMD18-T vector (TaKaRa, Japan) and subsequently verified by Sanger sequencing (Sangon Biotech, China). All primer sequences used in this study are listed in Supplementary Table 2.

### Plasmid construction and production of recombinant proteins

All eukaryotic expression and luciferase reporter plasmids were constructed using the ClonExpress II One Step Cloning Kit (Vazyme). Specifically, the ORFs of CaPGRP-L1, L2 and -S were cloned into the *EcoR* I/*BamH* I sites of p3XFLAG-CMV-14 vector to generate p3XFLAG-CaPGRP-L1, p3XFLAG-CaPGRP-L2, and p3XFLAG-CaPGRP-S plasmids expressing C- terminal 3 × FLAG-tagged fusion proteins. Meanwhile, the ORFs of snakehead p65, RelB, and c-Rel were inserted into the *Xba* I/*Hind* III sites of pcDNA3.1 to produce pcDNA3.1-p65, pcDNA3.1-RelB, and pcDNA3.1-c-Rel plasmids expressing MYC-tagged proteins. For subcellular localization studies, ORFs of snakehead PGRPs were cloned into the *EcoR* I /*Kpn* I sites of pCMV-C-EGFP vector to create GFP fusion constructs. Additionally, the region of 1500 nucleotides upstream from the start codon of each CaPGRP gene were inserted into the *Kpn* I/*Xho* I sites of pGL3-Basic vector, creating pGL3-CaPGRP-L1, pGL3-CaPGRP-L2, and pGL3-CaPGRP-S luciferase reporters. The commercial pNF-κB-luc reporter (D2206) was purchased from Beyotime. All primers used for these constructions are listed in Supplementary Table 2.

To obtain the recombinant protein of snakehead PGRPs, the constructed eukaryotic expression plasmids p3XFLAG-CaPGRP-L1, p3XFLAG-CaPGRP-L2, p3XFLAG-CaPGRP-S along with the empty vector p3XFLAG-CMV-14 as control were separately transfected into HEK293T cells using Lipofectamine 2000 Transfection Regent (Invitrogen) according to the manufacturer's protocol. After 48 h post-transfection (hpt), recombinant protein expression in cell lysates was verified by Western blot analysis with a mouse monoclonal anti-FLAG antibody (Dia-An Biotech). The transfected cell lysates were aliquoted and stored at −80 °C for subsequent experiments.

### Constitutive and induced expression analysis of snakehead PGRPs

To examine constitutive expression patterns of CaPGRP-L1, CaPGRP-L2 and CaPGRP-S, gill, trunk kidney, intestine, liver, brain, head kidney and spleen were collected separately from three healthy snakeheads for RNA extraction. For PGN-induced expression analysis, head-kidney leukocytes (HKLs) were isolated from three healthy snakehead following established protocols [22,23]. In brief, head kidneys were aseptically processed through a sterile nylon mesh in complete DMEM (Gibco) containing 100 U/mL penicillin, 100 μg/mL streptomycin (Thermo Fisher), and 12.5 U/mL heparin sodium (Sangon Biotech). The cell suspension was fractionated on a 34 % / 51 % Percoll gradient by centrifugation at 500 g for 30 min. HKLs collected from the obvious interface between the two Percoll density layers were resuspended in DMEM with 10 % FBS. Then, HKLs were plated in 24-well plates at a density of 1 × 10^6^ cells/well. After 2 h incubation at 28 °C, 5 % CO_2_, cells were stimulated with 20 μg/mL PGN (Invivogen, cat no. tlrl-pgns2) from *Staphylococcus aureus*, while an equal volume of PBS served as control. Cells were harvested at 3, 6, 12 and 24 h post-stimulation for RNA extraction. For quantitative real-time PCR (qRT-PCR) analysis, total RNA was reverse-transcribed into cDNA as previously described. qRT-PCR was performed using the CFX96 system (Bio-Rad) in 20 μL reaction volumes. The thermal cycling conditions consisted of an initial denaturation at 95 °C for 30 s, followed by 40 cycles of 95 °C for 10 s, 60 °C for 30 s, and 72 °C for 20 s. All reactions were performed in triplicate, with β-actin serving as the internal control. Relative gene expression was calculated using the 2^−ΔΔCT^ method. qRT-PCR primers were designed using Primer 5.0 and are listed in Supplementary Table 2.

### Expression analysis of snakehead PGRPs in cells by fluorescence microscopy

For confirmed the expression of snakehead PGRPs in cells, EPC cells were seeded in 24-well plates at a density of 2 × 10⁵ cells/well and transfected with either pEGFP-CaPGRP-L1, pEGFP-CaPGRP-L2, pEGFP-CaPGRP-S, or the empty vector pCMV-C-EGFP using Lipofectamine 2000 (Invitrogen) according to the manufacturer's protocol. After 36 hpt, cells were fixed with 4 % paraformaldehyde (PFA) at room temperature for 30 min, followed by nuclear staining with 5 μg/mL DAPI (Sangon Biotech) in dark for 30 min. Fluorescence images were obtained using a Zeiss fluorescence microscope.

### PGN-binding activity analysis

Recombinant snakehead PGRPs from transfected HEK 293T cell lysates were assessed for PGN-binding activity according to methods described in previous studies [22,36]. Briefly, HEK293T cells were seeded into 6-well plate and transfected with p3XFLAG-CaPGRP-L1, -L2, -S, pCMV-C-EGFP plasmid or the empty vector p3XFLAG-CMV-14 using Lipofectamine 2000 (Invitrogen). At 48 hpt, proteins were extracted from cells using lysis buffer supplemented with protease inhibitor cocktail (Roche). For the binding assay, 40 μg PGN was incubated respectively with protein extracts from p3XFLAG-CaPGRP-L1, -L2, -S, pCMV-C-EGFP or p3XFLAG-CMV-14 transfected cells in 500 μl TBS buffer (50 mM Tris–HCl, 50 mM NaCl, 10 μM ZnCl₂, pH 7.5) at 4 °C for 6 h with gentle shaking. Bound and unbound proteins were separated by centrifugation at 14,000 g for 15 min. The cell pellets were washed six times with TBS buffer. Then the input proteins indicating by “Free” protein and PGN-bound proteins were resolved by boiling 2 × SDS-polyacrylamide gelelectrophoresis (SDS-PAGE) loading buffer and detected by immunoblotting using anti-FLAG or anti-GFP antibodies (Dia-An Biotech).

### Amidase activity analysis

The amidase activity of snakehead PGRPs was examined using established methods with optimization [22,36,37]. In short, 40 mg PGN was incubated with 50 μg recombinant protein derived from transfected HEK 293T cell lysates, including p3XFLAG-CaPGRP-L1, p3XFLAG-CaPGRP-L2, p3XFLAG-CaPGRP-S, or p3XFLAG control vector, in Tris buffer (20 mM Tris–HCl, 150 mM NaCl, pH 7.2) with or without 10 μM ZnCl₂. Absorbance at 540 nm (OD₅₄₀) was measured at 10 min intervals for 120 min using a microplate reader.

### Antibacterial activity of PGRPs against *A. hydrophila*

The antibacterial activity of snakehead PGRPs against *A. hydrophila* was performed according to published methods with modification [37,38]. Briefly, EPC cells were seeded in 6-well plates at the density of 1 × 10^6^ cells/well and transfected with p3XFLAG-CaPGRP-L1, -L2, -S, or p3XFLAG-CMV-14 as control using Lipofectamine 2000 (Invitrogen). Following 48 h incubation at 37 °C, cells were infected with *A. hydrophila* (MOI = 10, 2 × 10⁷ CFU/mL) for 1 h. After being washed with DMEM for 3 times, extracellular bacteria were eliminated by 3 h treatment with DMEM containing 10 % FBS and 100 μg/mL gentamicin. Cells were then washed 4 times with DMEM and lysed in 500 μL PBS with 1 % Triton X-100 (20 min, RT). The number of bacteria was calculated by plate colony counting method.

### Dual-luciferase reporter assay

To analyze whether snakehead PGRPs could induce NF-κB signaling, HEK293T cells were seeded in 24-well plates at the density of 1 × 10^5^ cells/well and co-transfected with 300 ng pNF-κB-Luc, 30 ng pRL-TK and increasing concentrations (0, 100, 200, 300 ng) of p3XFLAG-CaPGRP-L1, -L2, -S or empty vector p3XFLAG-CMV-14. 48 hpt, the luciferase activity was measured using a dual-luciferase reporter assay system. Data were normalized to Renilla luciferase activity and calculated from three independent experiments performed in triplicate.

For assessing luciferase activity of snakehead PGRP proximal promoter reporters, EPC cells were seeded in 24-well plates at 1 × 10⁵ cells/well and co-transfected with 300 ng pGL3-CaPGRP-L1, -L2, -S, 30 ng pRL-TK, and 300 ng pcDNA3.1-p65, pcDNA3.1-RelB, pcDNA3.1-c-Rel, or empty vector pcDNA3.1. Luciferase activity was detected at 48 hpt using the same method described above.

### Statistical analysis

For tissue distribution data, independent samples *t*-test and One-way ANOVA with Duncanʼs test were used for statistical analysis. Additionally, Statistical significance of the amidase activity data was calculated using two-way ANOVA followed by Tukey's multiple comparisons test. Statistical analyses of qRT-PCR and dual-Luciferase reporter assay data were performed using Studentʼs *t*-test in SPSS 20.0. Significant differences were denoted as * *P* < 0.05, ** *P* < 0.01, and *** *P* < 0.001. Data from three independent experiments were presented as mean ± standard error (SE).

## Results

### Identification and sequence analysis of CaPGRP-L1, CaPGRP-L2 and CaPGRP-S

**Fig. 1:**
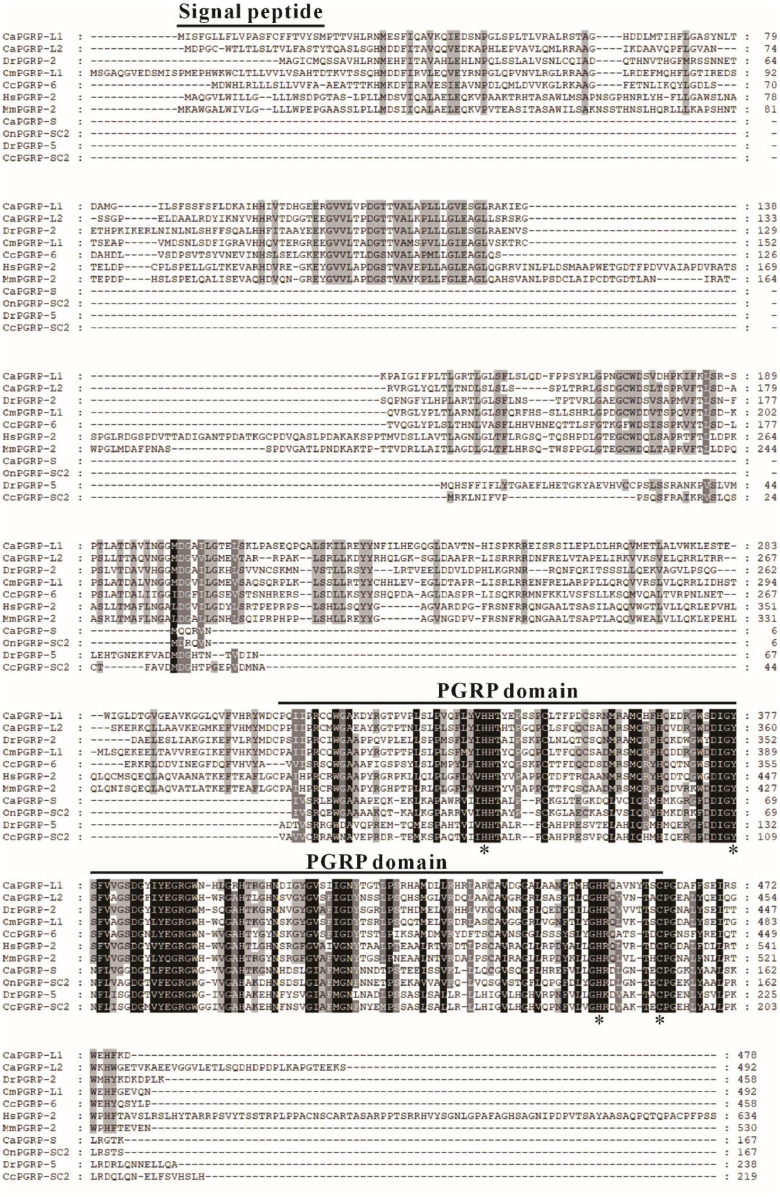
. Multiple sequence alignment of snakehead CaPGRP-L1, -L2 and -S with vertebrate orthologs. Amino acid sequences were aligned using ClustalX 2.1 and were edited with GeneDoc software. Black, dark grey, and light grey represent 100 %, 80 %, and 60 % conservation, respectively. The putative signal peptide and peptidoglycan recognition (PGRP) domains are indicated in solid lines above alignment. The Zn^2+^-binding amino acids essential for amidase activity are marked with asterisks below the sequences. The numbers on the right indicate the number of amino acids. Accession numbers for all PGRP sequences are provided in Supplementary Table 1.

**Fig. 2:**
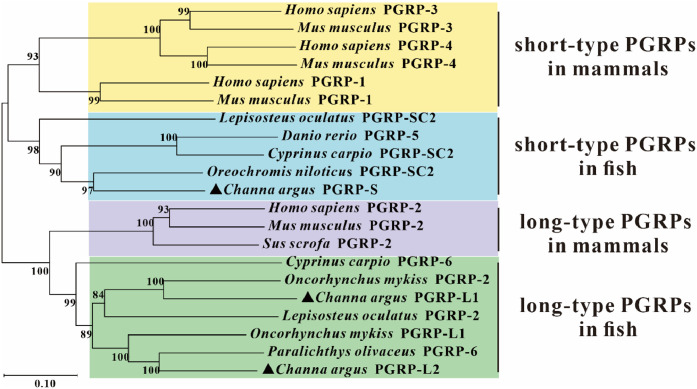
Phylogenetic analysis of snakehead CaPGRP-L1, -L2, and -S with mammalian and other fish PGRPs. The neighbor-joining (NJ) tree, based on deduced amino acid sequences with signal peptides, was generated in MEGA X software with 2000 bootstrap replicates (values shown at branch nodes). Snakehead PGRPs are marked by black triangles. The GenBank accession numbers of PGRP sequences are listed in Supplementary Table 1.

Through genome-wide screening, two long-type PGRP genes, CaPGRP-L1 and CaPGRP-L2, and one short-type PGRP gene, CaPGRP-S were identified in the snakehead genome. The entire ORF of CaPGRP-L1, -L2 and -S were 1437 bp, 1479 bp and 504 bp in length, encoding proteins of 478, 492 and 137 amino acids, respectively. The theoretical molecular mass of CaPGRP-L1, -L2 and -S mature peptide was 53.6 kDa, 54.21 kDa and 18.35 kDa. Multiple sequence alignment demonstrated that all three PGRPs contain a conserved PGRP domain with the characteristic Zn²⁺-binding residues (His340, Tyr408, His452 and Cys461 in Ca PGRP-L1, His333, Tyr360, His435 and Cys443 in Ca PGRP-L2, His34, Tyr69, His143 and Cys151 in Ca PGRP-S) (Fig. 1 and Supplementary Fig. 1). In addition, the CaPGRP-L1 and CaPGRP-L2 were predicted to be secreted proteins as the presence of putative signal peptide at the N-terminal region, but this is not the case for CaPGRP-S (Fig. 1 and Supplementary Fig. 1). To understand the evolutionary relationship among PGRPs from mammal and fish, a Neighbor-joining (NJ) tree was constructed using deduced protein sequences (Fig. 2). As shown in Fig. 2, all vertebrate long-type PGRPs formed a major clade, within which CaPGRP-L1, CaPGRP-L2 and other fish long-type PGRPs grouped together to form a distinct subclade with high bootstrap support (Fig. 2). Similarly, phylogenetic analysis showed that short-type PGRPs from all vertebrates were grouped into another large clade, with CaPGRP-S and other fish short-type PGRPs forming a distinct, well-supported subclade (Fig. 2).

### Expression analysis of CaPGRP-L1, CaPGRP-L2 and CaPGRP-S

**Fig. 3:**
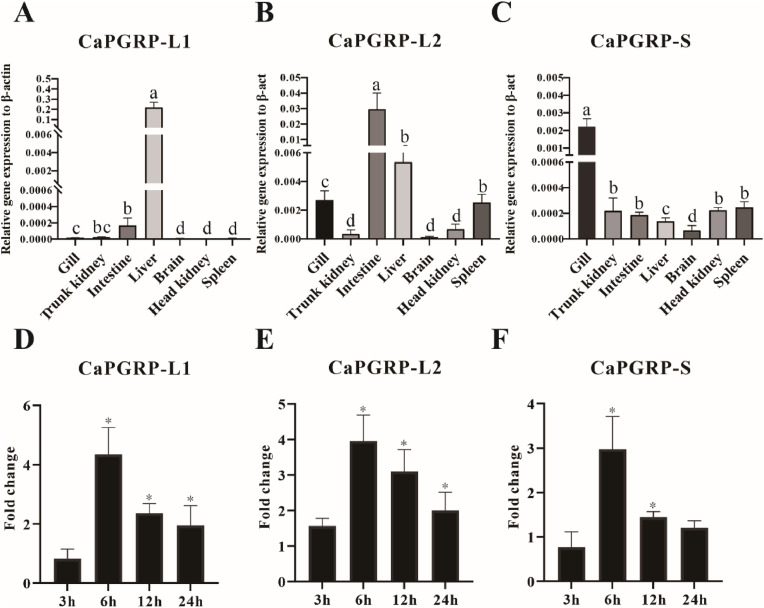
Tissue distribution and induced expression of snakehead PGRPs. The constitutive expression of CaPGRP-L1(A), CaPGRP-L2 (B) and CaPGRP-S (C) in different tissues/organs of healthy snakehead, including gill, trunk kidney, intestine, liver, brain, head kidney and spleen, was measured by qRT-PCR. Data were normalized against the level of β-actin and presented as mean ± standard error (SE) from three independent experiments performed in triplicates. The different letters (a-d) represent significant differences according to Duncan's multiple range test (*P* < 0.05). Expression of CaPGRP-L1 (D), CaPGRP-L2 (E) and CaPGRP-S (F) in PGN-induced HKLs at 3, 6, 12, and 24 h post-stimulation. Fold changes are shown relative to PBS control (mean ± SE). Significant differences are indicated by asterisks (*P < 0.05).

The mRNA expression profiles of the three snakehead PGRP genes of were detected by qRT-PCR across multiple organs / tissues, including gill, trunk kidney, intestine, liver, brain, head kidney, and spleen (Fig. 3A-C). qRT-PCR analysis revealed ubiquitous expression of CaPGRP-L1, -L2 and -S in all examined organs / tissues, albeit with distinct tissue-specific pattern (Fig. 3A-C). In detail, both CaPGRP-L1 and CaPGRP-L2 showed predominant expression in intestine and liver, but CaPGRP-L1 exhibited moderate expression in brain, spleen and head kidney, and CaPGRP-L2 had lower transcript levels in trunk kidney, brain and head kidney (Fig. 3A and B). In contrast, CaPGRP-S showed highest expression in gill (Fig. 3C). Following PGN stimulation, all three genes showed significant induction in HKLs at 6, 12 and 24 h, peaking at 6 h (Fig. 3D-F).

**Fig. 4:**
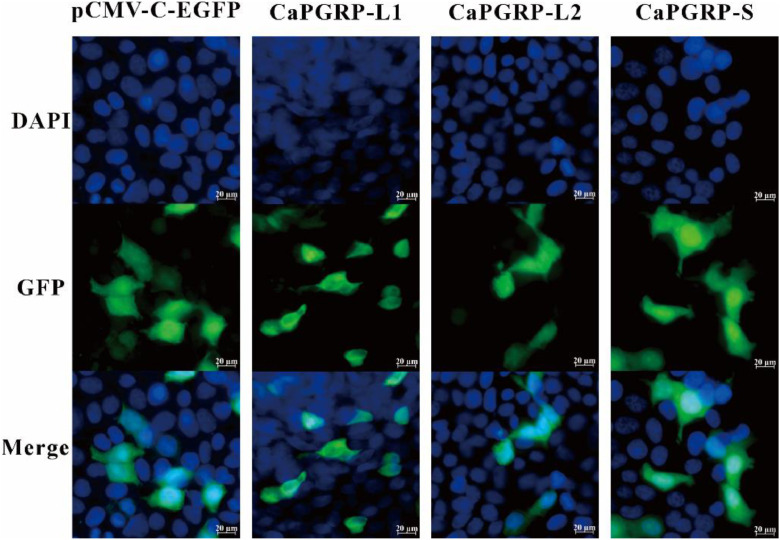
Expression analysis of CaPGRP-L1, -L2, and -S in EPC cells. Cells transfected with pEGFP-CaPGRP-L1, -L2, -S or control pCMV-C-EGFP for 36 h were stained with DAPI and imaged using a fluorescence microscope.

To confirmed the intracellular expression of CaPGRP-L1, CaPGRP-L2 and CaPGRP-S, the eukaryotic expression vectors of PGRP genes containing GFP-tagged were constructed in this study. Fluorescence microscopy revealed that all three PGRP proteins were successfully expressed in both the nucleus and cytoplasm (Fig. 4).

### PGN-binding activity of recombinant CaPGRP-L1, CaPGRP-L2 and CaPGRP-S

To obtain recombinant snakehead PGRP proteins, eukaryotic expression plasmids were constructed. Subsequently, CaPGRP-L1, -L2 and -S fused with a FLAG tag were expressed in HEK293T cells and analyzed by Western blot. The results revealed specific bands corresponding to CaPGRP-L1 (55–70 kDa), CaPGRP-L2 (55–70 kDa), and CaPGRP-S (15–25 kDa) in lysates from cells transfected with pCaPGRP-L1-FLAG, pCaPGRP-L2-FLAG, and pCaPGRP-S-FLAG. These observed molecular weights were slightly higher than their predicted sizes of 53.05 kDa, 54.21 kDa, and 18.34 kDa for CaPGRP-L1, -L2, and -S, respectively, which is likely attributable to the additional FLAG tag and potential post-translational modifications (Supplementary Fig. 2A). Specifically, p3XFLAG-CaPGRP-L2 showed higher transfection efficiency than -L1 and -S, likely leading to greater CaPGRP-L2 protein expression compared to -L1 and -S (Supplementary Fig. 2B).

**Fig. 5:**
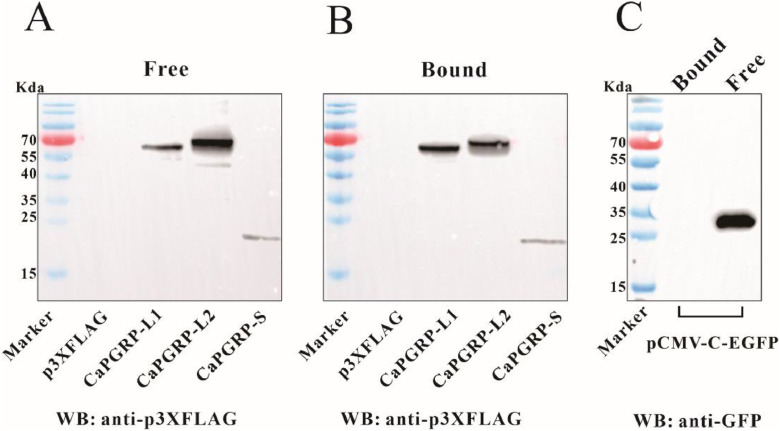
PGN-binding activity of snakehead PGRPs. (A-C) Lysates from HEK293T cells transfected with p3XFLAG-CaPGRP-L1, p3XFLAG-CaPGRP-L2, p3XFLAG-CaPGRP-S, pCMV-C-EGFP or the empty vector p3XFLAG were incubated with *S. aureus* PGN. Free proteins were isolated from bound proteins by centrifugation, and the pelleted bound fraction was washed six times. Protein samples were analyzed by SDS-PAGE and detected by immunoblotting using anti-FLAG (A, B) or anti-GFP (C) antibodies. Specific binding bands were detected for CaPGRP-L1, -L2 and -S, while control samples showed no binding activity. In the figure, ‘Free’ represents the supernatant while ‘Bound’ indicates the washed pellet.

The PGN-binding capacity of recombinant proteins was then evaluated. Cell lysates containing FLAG-tagged CaPGRPs were incubated with PGN, along with control lysates from cells transfected with either empty p3XFLAG or pCMV-C-EGFP vectors. As shown in Fig. 5, Western blot analysis demonstrated that CaPGRP-L1, -L2 and -S bound specifically to PGN, whereas no binding was detected in the empty p3XFLAG-CMV-14 or pCMV-C-EGFP controls.

### Zn^2+^-dependent amidase activity of recombinant CaPGRP-L1, CaPGRP-L2 and CaPGRP-S

**Fig. 6:**
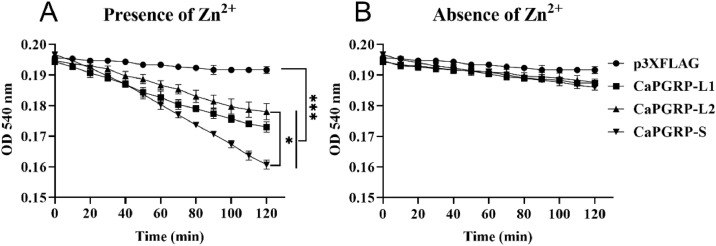
Amidase activity of CaPGRP-L1, -L2 and -S in degrading PGN from *S. aureus*. The lysates of HEK293T cells transfected with p3XFLAG-CaPGRP-L1, -L2, -S, and the empty vector p3XFLAG were incubated with PGN in the presence (A) or absence (B) of Zn^2+^, and the OD_540_ nm was recorded every 10 min during a 120 min period. Data represent mean ± SE (*n* = 3). Statistical significance was assessed using two-way ANOVA followed by Tukey's multiple comparisons test, with significance levels set at * *P* < 0.05 and *** *P* < 0.001.

Based on the conservation of four residues required for Zn^2+^-binding and amidase activity [39,40], CaPGRP-L1, -L2 and -S were hypothesized to exhibit amidase activity. To test, the recombinant snakehead PGRP proteins, as indicated above, were incubated with PGN, using empty p3XFLAG vector as control. In the presence of Zn²⁺, the absorbance of Tris-ZnCl₂ buffer containing CaPGRP-L1, CaPGRP-L2, or CaPGRP-S decreased significantly, confirming that all three PGRP proteins possess PGN-lytic activity (Fig. 6A). However, the activity was markedly reduced in the absence of Zn²⁺ (Fig. 6B). Furthermore, the PGN degradation activity of CaPGRP-S was significantly greater compared to CaPGRP-L2 (*P* < 0.05) (Fig. 6A). In contrast, PGN degradation was not observed in control (Fig. 6A and B). These results demonstrated that all three snakehead PGRP proteins show Zn^2+^-dependent amidase activity.

### Antibacterial activity of recombinant CaPGRP-L1, CaPGRP-L2 and CaPGRP-S proteins against *A. hydrophila*

**Fig. 7:**
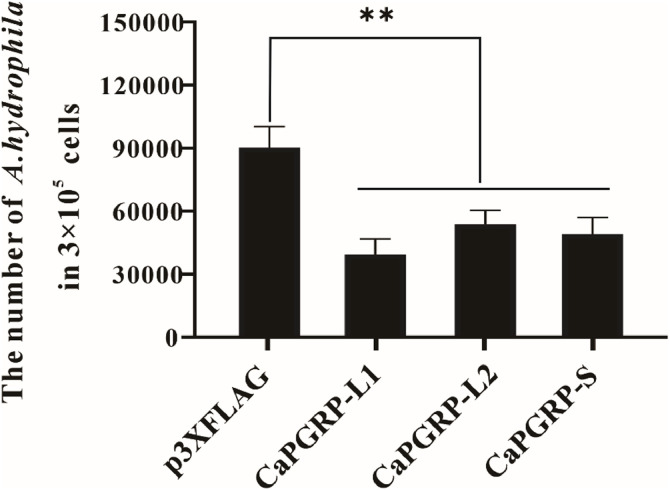
Antibacterial activity of snakehead PGRPs against *A. hydrophila* infection. EPC cells transfected with p3XFLAG-CaPGRP-L1, -L2, -S, and the empty vector p3XFLAG were infected with *A. hydrophila* (MOI = 10). Bacterial counts were determined at 1 h post-infection followed by 3 h gentamicin treatment (100 μg/mL) to eliminate extracellular bacteria. Data were expressed as mean ± SE, and ** indicating *P* < 0.01.

To evaluate the antibacterial potential of recombinant snakehead PGRPs, EPC cells transfected with pCaPGRP-L1-FLAG, pCaPGRP-L2-FLAG, pCaPGRP-S-FLAG, or empty p3XFLAG vector as control, then challenged with *A. hydrophila*. Plate counting analysis revealed significant reduced number of *A. hydrophila* in cells overexpressing CaPGRP-L1, CaPGRP-L2, or CaPGRP-S compared to empty vector-transfected control cells (Fig. 7), demonstrating the antibacterial activity of these recombinant PGRP proteins.

### Activation of NF-κB signaling by CaPGRP-L1, CaPGRP-L2 and CaPGRP-S

**Fig. 8:**
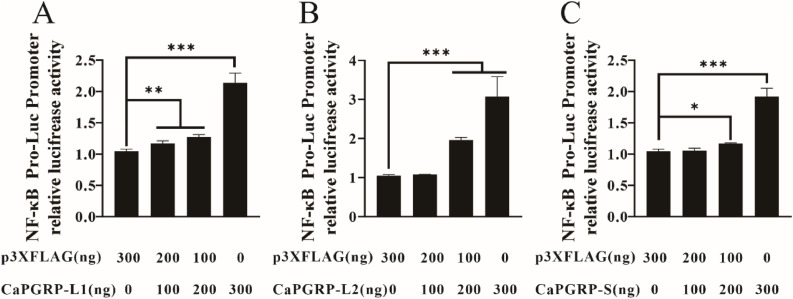
Regulation of NF-κB signaling by snakehead PGRPs. HEK293T cells were co-transfected with pNF-κB-luc, pRL-TK and increasing doses (0, 100, 200, 300 ng) of p3XFLAG-CaPGRP-L1 (A), -L2 (B), -S (C), or empty vector p3XFLAG. The luciferase activity was measured at 48 h post-transfection and normalized to Renilla luciferase. Data are presented as mean ± SE (*n* = 3). Significant difference is indicated by asterisks, * *P* < 0.05, ** *P* < 0.01 and *** *P* < 0.001.

In teleost fish and mammals, PGRPs may be able to activate the transcription factor NF-κB, leading to the expression of AMPs [22,[41], [42], [43]]. To investigate whether snakehead PGRPs are involved in NF-κB signaling pathway, the effect of CaPGRP-L1, L2 and -S was examined on NF-κB activation using a luciferase reporter system in HEK293T cells. The dual-luciferase reporter assay revealed a dose-dependent activation of the NF-κB reporter plasmid (pNF-κB-luc) by all three PGRPs (Fig. 8A-C). These findings demonstrate that CaPGRP-L1, -L2 and -S can promote NF-κB transcriptional activity in HEK293T cells.

### CaPGRP-L1, CaPGRP-L2 and CaPGRP-S promoter and its activity regulated by NF-κB

**Fig. 9:**
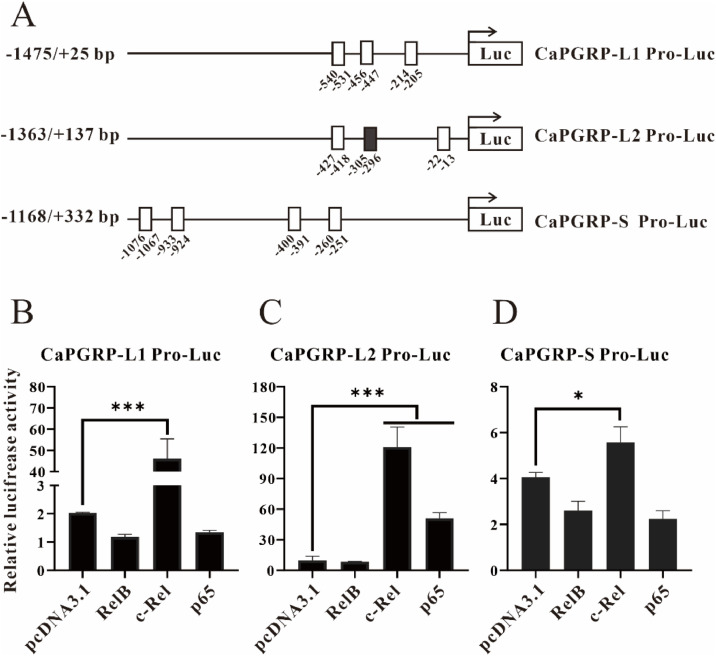
Regulation of CaPGRP-L1, -L2, -S proximal promoter reporter plasmids by NF-κB transcription factors. (A) Schematic representation of the luciferase promoter constructs for snakehead PGRPs. The 1500 bp upstream regions from the start codon of CaPGRP-L1, CaPGRP-L2, and CaPGRP-S genes were cloned into the pGL3-Basic luciferase reporter vector to generate promoter-driven reporter constructs. White and black boxes indicate putative NF-κB binding sites corresponding to the consensus sequences 5′-GGGRNNYNNN-3′ and 5′-GGGRNNYYCC-3′, respectively. The transcription initiation site is designated as +1, with numbers below denoting the positions of kB sites. EPC cells were co-transfected with promoter reporter constructs pGL3-CaPGRP-L1 (B), -L2 (C) or -S (D) and pRL-TK were transfected together with expression vectors for pcDNA3.1-p65, -RelB, -c-Rel or empty vector pcDNA3.1, respectively. The luciferase activity was measured at 48 h post-transfection and normalized to Renilla luciferase activity. Data are presented as mean ± SE (*n* = 3). Significant difference is indicated by asterisks, * *P* < 0.05, ** *P* < 0.01 and *** *P* < 0.001.

The transcription factor NF-κB can regulate gene transcription by binding to the κB site (consensus sequence 5′-GGGRNNYYCC-3′ or 5′-GGGRNNYNNN-3′, R, Y, and N represent purine, pyrimidine, and any nucleotide base, respectively) in promoter regions [44]. Several potential NF-κB binding sites were predicted in the proximal promoter regions of CaPGRP-L1, -L2, and -S (Fig. 9A and Supplementary text 1). Notably, only CaPGRP-L2 promoter contained a κB motif matching the consensus sequences 5′-GGGRNNYYCC-3′, while all other predicted κB sites conformed to the less strict motif 5′-GGGRNNYNNN-3′ (Fig. 9A and Supplementary text 1). To investigate the regulatory effects of NF-κB subunits on PGRP promoter activity, the eukaryotic expression plasmid pcDNA3.1-p65, pcDNA3.1-RelB, pcDNA3.1-c-Rel or the empty vector pcDNA3.1 was transfected separately in combination with snakehead PGRP promoter-driven luciferase reporter constructs and the internal control plasmid pRL-TK into EPC cells. The results of dual-luciferase reporter assays demonstrated that c-Rel significantly enhanced the luciferase activities of pCaPGRP-L1-luc, pCaPGRP-L2-luc and pCaPGRP-S-luc reporter plasmids (Fig. 9B, C and D). Moreover, p65 also increased significantly the luciferase activity of pCaPGRP-L2-luc (Fig. 9C). In contrast, RelB had no detectable effect on any PGRP promoter constructs (Fig. 9B-D). These findings suggest that NF-κB might play a role in regulating the expression of PGRPs.

## Discussion

PGRPs represent a crucial class of pattern recognition receptors (PRRs), and play pivotal roles in antibacterial immune responses as well as in modulating inflammatory processes across a diverse range of species [2]. In the present study, two long PGRPs (CaPGRP-L1 and CaPGRP-L2) and one short PGRP (CaPGRP-S) were cloned and characterized in snakehead. Their roles in PGN recognition, amidase activity, NF-κB signaling activation, antimicrobial activity were analyzed. The activation of PGRP promoter-driven reporter plasmids by c-Rel and p65 was also revealed in the present study.

PGRPs in the snakehead share conserved structural features as observed in mammals and teleost fish, in terms of characteristic PGRP domain containing highly conserved Zn²⁺-binding residues which are essential for amidase activity [19,21,39,40,45]. The long PGRPs, CaPGRP-L1 and -L2 were clustered with other long PGRPs from mammals and fish, while CaPGRP-S was grouped with short PGRPs in these species. The evolutionary relationship of PGRPs may suggest their functional conservation. Indeed, functional similarities were observed between snakehead PGRPs and those in mammals and other fish, including PGN-binding capacity, amidase activity, and antimicrobial properties. Collectively, these results demonstrate evolutionary conservation of both structural features and functional properties in snakehead PGRPs.

In terms of expression patterns, PGRPs exhibit remarkable tissue-specific constitutive expression, with particularly high levels in liver, spleen, intestine and gill [14,37,46,47]. Moreover, PGRPs can respond to the stimulation by bacterial pathogens [7,24,48]. In this study, CaPGRP-L1, -L2 and -S were expressed in all organs/tissues investigated, and CaPGRP-L1 had relatively high expression level in liver, being similar to mammalian PGLYRP2, with persistent expression and secretion in liver [13]. In addition, the PGRPs of snakehead also had relatively high expression levels in immune-related organs. On the other hand, CaPGRP-L1 and CaPGRP-L2 were highly expressed in intestine, whereas CaPGRP-S showed the highest expression in the gill. Intestine and gills are mucosal immune sites which can serve as the first line of defense against pathogen invasion [49]. Notably, PGRP-LB can modulate gut inflammatory responses [50]. Similarly, it may imply that PGRPs in snakehead may be involved in regulating mucosal immunity. In addition, Following PGN stimulation, all three PGRPs exhibited significant transcriptional upregulation HKLs, peaking at 6 h post-stimulation. This rapid induction pattern indicates their potential role in initiating immediate innate immune response against pathogenic invasion.

Functionally, PGRPs can specifically recognize bacterial PGN, thereby initiating innate immune responses [6]. In this study, as reported in other animals, recombinant CaPGRP-L1, -L2 and -S proteins all exhibited PGN-binding ability and Zn²⁺ dependent amidase activity [15,22,27,46]. Moreover, sequence analysis revealed that all three snakehead PGRPs contain a conserved Zn²⁺-binding site with two His, one Tyr, and one Cys, a feature that indicates they belong to catalytic PGRPs. Studies have shown that insect catalytic PGRPs primarily function as bactericides or immune regulators to prevent excessive inflammation [6,51]. Similarly, zebrafish catalytic PGRPs can also suppress immune responses to bacterial infections, thereby preventing sustained inflammatory conditions [28,29,52]. This suggests that snakehead PGRPs likely play analogous roles in maintaining immune homeostasis, making them of interest for subsequent research. Notably, CaPGRP-S exhibited the highest amidase activity among the three tested PGRPs. This observation aligns with previous studies reporting remarkable amidase activity in other fish PGRPs, including orange-spotted grouper PGRP-S and Nile tilapia PGRP-SC2 [21,22]. In contrast, PGRP2 in spotted sea bass (*Lateolabrax maculatus*) demonstrated markedly stronger activity compared to PGRP-SC2 and PGRP-L2 [24]. So far, the antibacterial functions of fish PGRPs have been extensively characterized. For instance, spotted sea bass PGRPs inhibit the survival and growth of *S. aureus, Vibrio harveyi*, and *Edwardsiella tarda*, while rock bream PGRP2 suppresses *Edwardsiella piscicida, Streptococcus iniae*, and *Streptococcus parauberis* [24,53]. Similarly, grass carp PGRP6 and orange-spotted grouper PGRPs exhibit inhibitory effects against *E. tarda* [42]. In our study, the obvious inhibitory effect on the growth of *A. hydrophila* in cells overexpressing PGRPs further supports their potential antimicrobial role, a finding that requires further investigation to elucidate the underlying mechanisms.

In innate immunity, PGRP-mediated pathogen recognition has been known to activate NF-κB signaling pathway, thereby inducing the production of antimicrobial peptides (AMPs), which are recognized as crucial components of innate immunity and provide the first line of defense against diverse pathogens [11]. Similar to the findings in the orange-spotted grouper, spotted sea bass, and grass carp [22,23,42,43], CaPGRP-L1, -L2 and -S were found to increase the luciferase activity of the NF-κB promoter. Moreover, studies in mice have demonstrated that NF-κB transcription factors can activate PGRP-S transcription through binding κB motifs in the promoter region [10]. Intriguingly, it was also revealed in the present study that NF-κB subunits (c-Rel and p65) enhanced the luciferase activity of snakehead PGRP promoter reporter plasmids, suggesting a positive feedback regulation between PGRPs and NF-κB. Previous studies have established that κB site sequences display considerable variation, which may result in differential NF-κB recognition affinity [44]. In current study, luciferase assays demonstrated significantly stronger NF-κB-mediated activation of the CaPGRP-L2 promoter compared to CaPGRP-L1 and CaPGRP-S. Notably, bioinformatics analysis identified a complete κB consensus site 5′-GGGRNWYYCC-3′ exclusively in the CaPGRP-L2 proximal promoter, whereas CaPGRP-L1 and CaPGRP-S contained only half-site κB motif 5′-GGGRNNYNNN-3′. Therefore, the differential NF-κB-mediated transcriptional activation of PGRP promoters is likely determined by their κB site variations, though the exact molecular mechanisms remain to be elucidated. Nonetheless, these results provide new clues for further exploration of the molecular mechanisms by which fish PGRP is involved in NF-κB signaling pathway.

In summary, three PGRP genes, including two long type, PGRP-L1 and -L2, and one short type, PGRP-S were cloned in snakehead. Their constitutive expression and induced expression in HKLs were examined. Furthermore, these PGRPs were found to have PGN-binding capacity and amidase activity, and these PGRPs can regulate NF-κB, and also be regulated transcriptionally through NF-κB, which provides a further clue for their transcriptional mechanism in immune response in fish.

## CRediT authorship contribution statement

**Zhen Yu Zhou:** Validation, Investigation, Data curation. **Wei Hao:** Validation, Software, Investigation. **Pin Nie:** Writing – review & editing, Supervision, Project administration, Funding acquisition. **Shan Nan Chen:** Validation, Data curation. **Jing Hou:** Writing – original draft, Supervision, Methodology, Formal analysis, Conceptualization.

## Declaration of competing interest

The authors declare that they have no known competing financial interests or personal relationships that could have appeared to influence the work reported in this paper.

## Data Availability

Data will be made available on request.

## Appendix Supplementary materials

Image, application 1
