# Supplementary material for: Expression and functional characterization of three peptidoglycan recognition proteins, PGRP-L1, PGRP-L2 and PGRP-S in snakehead (Channa argus)
Source: Comp Immunol Rep. 2025 Sep 4;9:200248. doi: 10.1016/j.cirep.2025.200248 (PMC12745986; doi:10.1016/j.cirep.2025.200248)
Supplement: Supplementary file 1 [file mmc1.docx]

**Supplementary table 1.** The GenBank accession numbers of PGRPs used for multiple sequence alignment and phylogenetic tree construction.

| **Species** | **Name** | **GenBank accession** |
| --- | --- | --- |
| *Channa argus* | PGRP-L1 | KAF3687498.1 |
|  | PGRP-L2 | KAF3700593.1 |
|  | PGRP-S | KAF3692979.1 |
| *Danio rerio* | PGRP-2 | NP_001038631 |
|  | PGRP-5 | NP_001037786 |
| *Lepisosteus oculatus* | PGRP-2 | XP_069051843 |
|  | PGRP-SC2 | XP_015196380 |
| *Oncorhynchus mykiss* | PGRP-L1 | XP_036797118 |
|  | PGRP-2 | XP_021423766 |
| *Paralichthys olivaceus* | PGRP-6 | XP_019961312 |
| *Cyprinus carpio* | PGRP-6 | XP_042575604 |
|  | PGRP-SC2 | XP_042631798 |
| *Oreochromis niloticus* | PGRP-SC2 | XP_003441739 |
| *Homo sapiens* | PGRP-1 | NP_005082 |
|  | PGRP-2 | NP_443122 |
|  | PGRP-3 | NP_443123 |
|  | PGRP-4 | NP_065126 |
| *Mus muscμlus* | PGRP-1 | NP_033428 |
|  | PGRP-2 | NP_067294 |
|  | PGRP-3 | NP_997130 |
|  | PGRP-4 | NP_997146 |
| *Sus scrofa* | PGRP-2 | NP_998903 |

**Supplementary table 2.** PCR primers used in this study

| Primer | Sequence 5'→3' | Purpose |
| --- | --- | --- |
| CaPGRP-L1F | ATGATTTCATTTGGACTGTTATTTC | ORF cloning |
| CaPGRP-L1R | TCAGTCTTTGAAGTGTTCCCAG |  |
| CaPGRP-L2F | ATGGATCCGGGCTGCTGGACAT |  |
| CaPGRP-L2R | TCAGGACTTCTCTTCCGTTCCTGGT |  |
| CaPGRP-SF | ATGCAGCAGAGAGTGAATATTGTTT |  |
| CaPGRP-SR | TTACTTAGTTCCTCTAAGTTTTGACAGT |  |
| β-actin-Q-F | CACTGTGCCCATCTACGAG | Real-time PCR |
| β-actin-Q-R | CCATCTCCTGCTCGAAGTC |  |
| GAPDH-Q-F(HEK293) | GATTTGGTCGTATTGGGCGC |  |
| GAPDH-Q-R(HEK293) | TTCCCGTTCTCAGCCTTGAC |  |
| CaPGRP-Q-L1F | CCCTCCTCACCCTGTCTAAC |  |
| CaPGRP-Q-L1R | AAACTGTATCCTATGTCGCTCC |  |
| CaPGRP-Q-L2F | CGTCTGGGATCTGATGGCT |  |
| CaPGRP-Q-L2R | GCTGGTGGCGGTAGTAGTCT |  |
| CaPGRP-Q-SF | TCACGACTCACTGGGAATAGC |  |
| CaPGRP-Q-SR | GTGCCCTAACAAAACAAACTCT |  |
| pEGFP-CaPGRP-L1F | TCCAAGCTTCTGCAGGAATTCGCCACCATGATTTCATTTGGACTGTT | Construction of eukaryotic expression vector |
| pEGFP-CaPGRP-L1R | GCCCTTGCTCACCATTCTAGAGTCTTTGAAGTGTTCCC |  |
| pEGFP-CaPGRP-L2F | TCCAAGCTTCTGCAGGAATTCGCCACCGCCACCATGGATCCGGGCTGCTGG |  |
| pEGFP-CaPGRP-L2R | GCCCTTGCTCACCATTCTAGAGGACTTCTCTTCCGTTCCTGGTGCT |  |
| pEGFP-CaPGRP-SF | TCCAAGCTTCTGCAGGAATTCGCCACCATGCAGCAGAGAGTGAATAT |  |
| pEGFP-CaPGRP-SR | GCCCTTGCTCACCATTCTAGACTTAGTTCCTCTAAGTT |  |
| p3XFLAG-CaPGRP-L1F | CCGGAATTCGCCACCATGATTTCATTTGGACTGTT |  |
| p3XFLAG-CaPGRP-L1R | CGCGGATCCGTCTTTGAAGTGTTCCCAG |  |
| p3XFLAG-CaPGRP-L2F | CCGGAATTCGCCACCATGGATCCGGGCTGCTGG |  |
| p3XFLAG-CaPGRP-L2R | CGCGGATCCGGACTTCTCTTCCGTTCCTGGTGCT |  |
| p3XFLAG-CaPGRP-SF | CCGGAATTCGCCACCATGCAGCAGAGAGTGAATA |  |
| p3XFLAG-CaPGRP-SR | CGCGGATCCCTTAGTTCCTCTAAGTTTTGACAG |  |
| pcDNA3.1-p65-F | AAACGGGCCCTCTAGACGCCACCATGGCTGGTGCCTATGGATG |  |
| pcDNA3.1-p65-R | TTTTTGTTCGGGCCCAAGCGGAAGGACTTTAAGTTGGGT |  |
| pcDNA3.1-RelB-F | AAACGGGCCCTCTAGACGCCACCATGAAAGACATGGACGTCAG |  |
| pcDNA3.1-RelB-R | TTTTTGTTCGGGCCCAAGTAGCGTCTCTTCTGTCTTCAC |  |
| pcDNA3.1-c-Rel-F | AAACGGGCCCTCTAGACGCCACCATGGCTGTGGCCGAACCAGCAA |  |
| pcDNA3.1-c-Rel-R | TTTTTGTTCGGGCCCAAGGTTGCCTTGCCGACCTGATTTG |  |
| pGL3-CaPGRP-L1F | ATTTCTCTATCGATAGGTACCAATGTTGTTCTTCTGTTGC | Construction of promoter reporter plasmid |
| pGL3-CaPGRP-L1R | GCTTACTTAGATCGCAGATCTCTTGGCTCTGCTGCTTGCTG |  |
| pGL3-CaPGRP-L2F | ATTTCTCTATCGATAGGTACCGGTCGCAAACAAACAAACAC |  |
| pGL3-CaPGRP-L2R | GCTTACTTAGATCGCAGATCTTCTGAGCGGCTCCGAACG |  |
| pGL3-CaPGRP-SF | ATTTCTCTATCGATAGGTACCAACTCCGTAAACACTTGG |  |
| pGL3-CaPGRP-SR | GCTTACTTAGATCGCAGATCTGGAGGCAGTGTTCTTCCAGTCT |  |

**Supplementary Figure 1.** Schematic diagram of the domains of PGRP protein in snakehead. The green boxes represent PGRP domains, while the red boxes denote signal peptides. The numbers below each box denoting their position within the amino acid sequence.


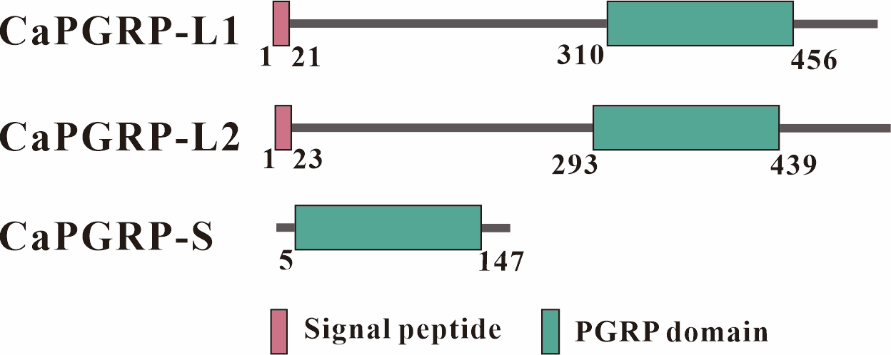


**Supplementary Figure 2.** Confirmation of the expression of CaPGRP-L1, CaPGRP-L2 and CaPGRP-S proteins in HEK293T cells. **A**. Cell lysates transfected with p3XFLAG-CaPGRP-L1, p3XFLAG-CaPGRP-L2, p3XFLAG-CaPGRP-S, and the empty vector p3XFLAG-CMV-14 as control. Then proteins eluted from the pellets were separated by SDS-PAGE and detected by Western blotting with the anti-FLAG antibody. **B**. Relative expression levels of CaPGRP-L1, CaPGRP-L2, and CaPGRP-S in HEK293T cells. Cells were seeded in 24-well plates at a density of 1 × 10⁵ cells/well and transfected with 250 ng of p3XFLAG-CaPGRP-L1, p3XFLAG-CaPGRP-L2, or p3XFLAG-CaPGRP-S plasmids. After 24 h, total RNA was extracted, reverse-transcribed, and subjected to qPCR to quantify target gene expression. Data were normalized to GAPDH levels and presented as mean ± standard error (SE) from three independent experiments performed in triplicate. Significant difference is indicated by *** *P* < 0.001.


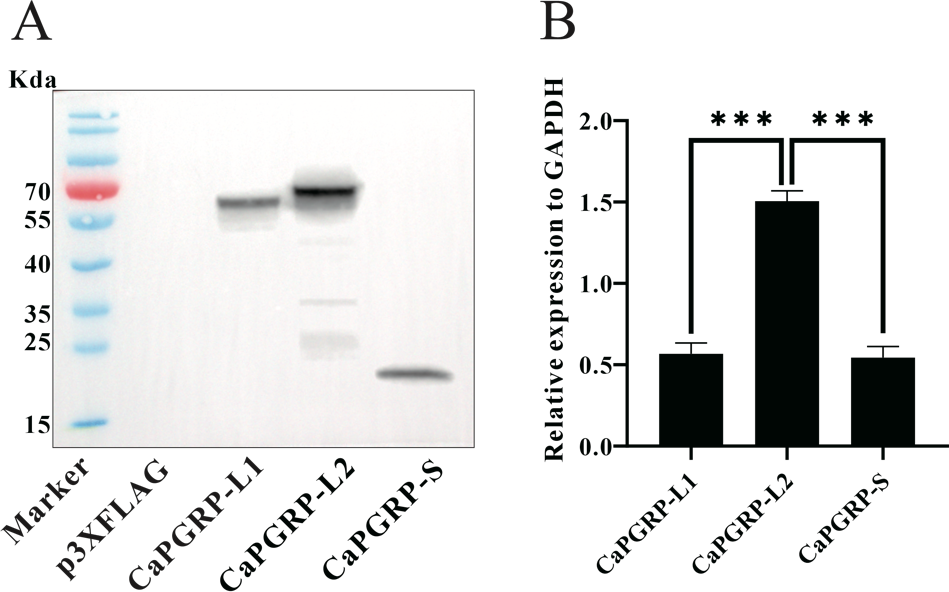


**Supplementary text 1.** Sequence analyses of the 5'-flanking regulatory regions of CaPGRP-L1, CaPGRP-L2, and CaPGRP-S genes. The 1.5 kb sequence upstream of the start codon was analyzed for NF-κB transcription factor binding sites. Potential κB motifs matching the consensus sequence 5'-GGGRNNYNNN-3' and 5'-GGGRNNYYCC-3' are highlighted in yellow and blue, respectively. The start codon (ATG) is boxed, and the putative transcription initiation site, derived from publicly available data, is marked in red.

>CaPGRP-L1 5' flanking regulatory sequence

AATGTTGTTCTTCTGTTGCATTATACTGCTGCCATTGCTGATTTATTTTCTTCCACACTATGCTTTTATCCTATGATCTTGAAAAACTAATATTAATTTTAATATCCCCACCCCCGCCCCTGATACCTGTGTGTACTGGCACCCACCTGATCGACATTGTCACCTTGGTTAACTATTGCTGAATTGGTGAAAGCGTTTTACAACAAGTGTGATAGTGATTACTAAGTCTGGCTTACACTGGCTCTACTTGCCTTAATAGTTTTCAGTGCTGACGTAGTGCTGAACAGATTATGATGCCATAATCACTGCAAATAGTTCATCATTAAATACACTTACACTGACTGCAATTCCTCCATTTCCTTTGATTAACCTCACTATGTAACTACTAGCCCCCACCCCTTCTTTACGAGGAGGTGTTATTAATTTCCTTCAAGTTCACGTCCTTGGGTAAATTAGAGTTAAAGATCCTTCAGGGGTCGCCACTTTGATTTGGCATAAGTTTTGGCATATGTGCTTCCTGTGGCAAAACTCCAACTTTTTTTATTTGCGCCTGGTGGAGTAAAATTTAAATCCCACTAAATGATTTTGCCTCTTGCAGCTGAGCCACTGAAATCTGCAATTGAACAACTGCACAACTGCACATTCATAAAACTTGTTTAAGTGCAATGCATAGAGTTAATAAGGAGGGGATATTGGTTTGATTTAGTGTTAAGTTAAAATGACTGCATGCTTATGTGGCCAGTTGGTGATAGTTTTTTCAACACTATTGTCTTTTATCAACATTTCCAATTAGGGCAGTTTTGTTTTTTGTGATTCTCCTCCATTTAAAGTTTTCATACAGTATCATAAATATTGACTAAGAGAGTTTCCTGACACTGGTGGATACAAAACAAGTGCACACGTCACATGAGATTAAACATTACCTAAAAATCTGCATCATCTCCCTCTTCAGTGGTGTTTATTCACTTCACTTTACTTCAAGAGATATAAATCGATCCAACAAAGGCAAAATGTCAGATGGGAGATGCACAAAAACCACAAAAAGATGCAATATAATGCAAAAATGACCAACAAGGACATAATCCTACACAAAGGAAACACAAAAGGACCAACTAACCCTAACAGTATGATTTAAATCAGACAAAATGTGACATAGAACAGAAAAAAAGACCAAAGAATGACATAAAAACAGGGGTATTGAGCTCTTCCTGTGTCTGTGCCCATTTTCTCAAAATCTGTTCATCATGAATGGGAAAACGCGGGGACTTTGTTTTGCCTGGCAGACTTTAAAATGTACTGTGTACTAGATCAATATTTGGAGTCATCTGTGTACAGAGAGTACTTACACAACTCCTCTGAGGTAGTTTAAACTTTCAGAAAACTTCCACATTGAGGATTATTGTGAAGATTCTTCCCTCATACCTGACTGTTTAAAGTGGTTTGTTCAATTGTGGTTCAATTAGAAAATATTTTTT**T**AAGGCAGCAAGCAGCAGAGCCAAGATG

>CaPGRP-L2 5' flanking regulatory sequence

GGTCGCAAACAAACAAACACAGGCCACATAGCGCCACCCAGTGCTGAATCAGCGGAGGTCCAAGTGGTAAAAACCATTCAACAGCAACTTCTCTTTCTGTGGCCAAAGGATTTTTTCATTAATTCAACAGGTCACAGTCTCAATTCACCAGGATGCTGAATCCAGCTGAGTCTGCAGACAGATGCAGTACAGAGCAAATGTTGAAGTCTGTGGGTTTTACTTACTACACACACATACACACATCTACACATTTTGCACAATTCTGTGTCCGTTTTTTACACAGTTAGTGCCTGGTTTTGGTTGGTTTGTCCCTTTTATATATACATTATTTTTAAGTTGTCCATTATTTTATCATGTTGCGTATTTGTTTGAGATAATTTTGTGTCTTTTAACTGAGACATAACTGGCCTGTGCCTTGTAAGGCTGGAAAGTAATCTAACCAGTTGAAATGTTACATCTAAGCTGTTAGTCAACACTTTCAGCACATCTTAAATGTTTCATGATGGCTCTCAGGTCTCTTCAGTCTGCAGCAGACCAGTCTCATGTCCCTGCTCCTGAGAAGCCCCCTCCTCCATGGCTGCCCCCACCAGGCTTCACCACAGAGCTGCTATTAGCAGGTGATGAGCAGTGTCTTCTGTCTGACCTGTGCACCGTAAAGCTGCTGAGCTGGTCGTCCAAACTTTAGTTTCGCTACACATTTTATATCCAGAATCATGTTTGCACAGGAAAAAAAAACCTCTTTACATTTAAAAATATTTAGTTCATCTGTTTTAGTCACTGCACCTCCCTCTATAGAACTTTATCATCACTGAGAACAGTAATAACTAATTTAACTCCTTTACATTCCCAGTTCCACTTCCTGTTCTTCCTGTTCTTGCTGTCACGTGACTCGGTGATCCTTGATCTGTGACATTCAATCTCAGCAGTTTGTGACACGGGATACTTCACCAAGGTCTGAGTGTGCACATGCAGCCCTCAGTGAAAATCAGATATGAATAAATCAGCATGCAGATGGGTTCCTGTCCCTGCTGCTCTGCACACACGTGATTCATGGTTTTGGGAGTTCCCCAGTTCTGTGTTTTCTGCACGTTAACGAGAACATTATTGCCTCAGAGAGGAGTGCCAATGATTTGTATAGCGATGCAGAGTTTGCTGCTGGCTGACCATATTCCCACCAGGTTTGTTTCACAAGCAGCAGCCACATAAACACTACTAAGATTTGTTGCAGAAGTTGTGAATTTTTAAAAAATATCCAGGTCAGTCACATCCACAGTGTGTGAGGCTGCAGGAAGACCCTCAAAGGTTAGGACAGAAGAATATACGCTGGCCACAGTGCAGATGCCCGTTTCCCACATTGCTCAGT**C**GGCCACACCACCTGATTAAACGCAGGCTTATCTACAGTATGTGTACAGCTGTTACATAACCAGCCACTGACGCTGCACGTCTATAAAAGTCAGCAACAACAAGGCAACAGGCGTTTGACGTTCGGAGCCGCTCAGAATG

>CaPGRP-S 5' flanking regulatory sequence

ACTCCGTAAACACTTGGCTTTGGCTGCGGACTGACGAGTAGTAGCATGTGCAGCAGCCTTATCAAAAAGTCAGTTTCAGGAGGTGTGGTCATGGGAAGCACACATTACCTTTGTTATTGAATGTGGATACATGTCACTGCAACAATCACAAGTAAATTTTTAACTGATTGTGCCGTCTTACAGATATTCAGCAGTTACCCAAGAGATTATTTGAAATAAACATGTTTGCAAAAAAGGGAATTTAAATATTCCATATCATGACAGAGAAACTGTGGGAGAGTAGATGATTGTAAGGTAGACGTTTAAGTATCATGTAGGAGGAATACAAGTGGAATAACCTTTAAACAATTAAGTATTTCACACCCTGAAGAGAGGCCACTTAGTGTTTGTTTTGCCAAAAGCATGGGTGTGTCTACTAATGTGAATAATTACAGAAGTATTTGTTACATGTTTGAGTAAACACTAGAGACGTGTCAAACAAGTGTGTATTTTATTCCAACAAAATACCTGAAAATTACAACTCTTAAAAAAAATAAAACAAAAGCCACATTGCTAATTTATAGTGCAGCCTGCAATTGTCAGTGTAATGCAGGCGTATGAATCAGGAACCAATAAAATACTTAGCACTTCACACTGGTACTTAAATGTAGTTGCGATGGATGAAAACAAGACTTTGGTGAACAAGACAATGTCAATGTGAACAGGCAATTTTTACTTTAGGTCTTTTTCTAAGAACTGCATCTCATGAGAACCAGTAGACAGTTAAAATTTGTTCCCCAATGTCTTGCTTTCTTCCATCGGTATCTGCATCATACAGTGAAATTAGACTAAATCTTAGGCTTTATTACAAAACGTTACCAAAACTATTAATCCATTCTAGTCACAAAATGGTGTCTGACATCATGTAAGGGACATCATAAAAGACAGAATCCAGGGTCTACAAAGAACTGTCTAGTAATGGAATCTATTATATTCACAGTGACCAGATTGATTGTATTTGGTTTCTGACTGATATGGTCAAAAAACAAAACAAAACAAAAAAGAAAACAAACCAAAAACCAATTTGGGCAACAGCTGGGAATATTTTTGAGTAGCATGTAGATGAAAATGATAGACCCGTGCTTTCACCAGCTTTACTCTGTCCAACGGTGTCCGCAGTGAGGGGCCG**T**GCAGACATAGTAGAGTCTCATAGCATCCTGTCAGGAGGAGGAAAATGTTTAATGTTTTACTTAAGTTTTATTTGCTTATTTTATCTTATGTGTATGGAGGGAAGCTTAAATTTTGTCTGTGTTTTGCAGCACTACAAATGTCTTAATTGTATACGTTTGGATGAAAGCAGACTATATTGCTAAAAGGTGAAAGCGACATTCTGCTGACCACGGTAAGCTGGTAAACTTATCCATATATAAACTGCACAAAAGACTTAAGGTCATGCACTGTATTTAATTACAGAGCTTACCTCAGCCTTCATACTGTGAGACTGGAAGAACACTGCCTCCGATG
